# Cytotoxic N-Methylpretrichodermamide B Reveals Anticancer Activity and Inhibits P-Glycoprotein in Drug-Resistant Prostate Cancer Cells

**DOI:** 10.3390/md20100597

**Published:** 2022-09-23

**Authors:** Sergey A. Dyshlovoy, Tobias Busenbender, Jessica Hauschild, Elena V. Girich, Malte Kriegs, Konstantin Hoffer, Markus Graefen, Anton N. Yurchenko, Carsten Bokemeyer, Gunhild von Amsberg

**Affiliations:** 1 Department of Oncology, Hematology and Bone Marrow Transplantation with Section Pneumology, Hubertus Wald Tumorzentrum—University Cancer Center Hamburg (UCCH), University Medical Center Hamburg-Eppendorf, Martinistrasse 52, 20246 Hamburg, Germany; 2 Martini-Klinik, Prostate Cancer Center, University Hospital Hamburg-Eppendorf, Martinistrasse 52, 20246 Hamburg, Germany; 3 Institute of High Technologies and Advanced Materials, Far Eastern Federal University, FEFU Campus, Ajax Bay 10, Russky Island, 690922 Vladivostok, Russia; 4 G.B. Elyakov Pacific Institute of Bioorganic Chemistry, Far-East Branch, Russian Academy of Sciences, Prospect 100 let Vladivostoku 159, 690022 Vladivostok, Russia; 5 Department of Radiotherapy & Radiation Oncology, Hubertus Wald Tumorzentrum—University Cancer Center Hamburg (UCCH), University Medical Center Hamburg-Eppendorf, Martinistrasse 52, 20246 Hamburg, Germany; 6 UCCH Kinomics Core Facility, Hubertus Wald Tumorzentrum—University Cancer Center Hamburg (UCCH), University Medical Center Hamburg-Eppendorf, Martinistrasse 52, 20461 Hamburg, Germany

**Keywords:** marine fungi, N-methylpretrichodermamide B, prostate cancer, cytotoxic activity, drug-resistance, p-glycoprotein, MAP kinases

## Abstract

N-methylpretrichodermamide B (NB) is a biologically active epidithiodiketopiperazine isolated from several strains of the algae-derived fungus *Penicillium* sp. Recently, we reported the first data on its activity in human cancer cells lines in vitro. Here, we investigated the activity, selectivity, and mechanism of action of NB in human prostate cancer cell lines, including drug-resistant subtypes. NB did not reveal cross-resistance to docetaxel in the PC3-DR cell line model and was highly active in hormone-independent 22Rv1 cells. NB-induced cell death was stipulated by externalization of phosphatidylserine and activation of caspase-3. Moreover, inhibition of caspase activity by z-VAD(OMe)-fmk did not affect NB cytotoxicity, suggesting a caspase-independent cell death induced by NB. The compound has a moderate p-glycoprotein (p-gp) substrate-like affinity and can simultaneously inhibit p-gp at nanomolar concentrations. Therefore, NB resensitized p-gp-overexpressing PC3-DR cells to docetaxel. A kinome profiling of the NB-treated cells revealed, among other things, an induction of mitogen-activated protein kinases JNK1/2 and p38. Further functional analysis confirmed an activation of both kinases and indicated a prosurvival role of this biological event in the cellular response to the treatment. Overall, NB holds promising anticancer potential and further structure–activity relationship studies and structural optimization are needed in order to improve its biological properties.

## 1. Introduction

Epithiodiketopiperazines (ETDPs) are a unique class of sulfur-containing diketopiperazine alkaloids, which have di-, tri-, and polysulfide bonds between the α- or α- and β-positions of two amino acid residues. ETDPs are characteristic metabolites of the fungi belonging to the *Penicillium* and *Trichoderma* genera [1]. Since the discovery of glioverin in 1982 [2], over 80 fungal-derived ETDPs have been reported. These compounds can be divided into three groups: glioverin-like, aspirochlorine-like and epicoccin-like molecules [1]. Glioverin-like metabolites contain the 1,2-oxazadecaline moiety in their structure, which is rather rare among natural compounds. These compounds were found in both terrestrial endophytic and marine endophytic fungi. Among others, this group includes glioverin [2], its methylated analog FA-2097 [3], halogenated derivatives [4,5], aspergillazines (the only ETDPs found in fungi *Aspergillus* genera) [6], and pretrichodermamides [7,8,9,10], some of which are also named adametizines [11] and outovirins [12].

**Figure 1:**
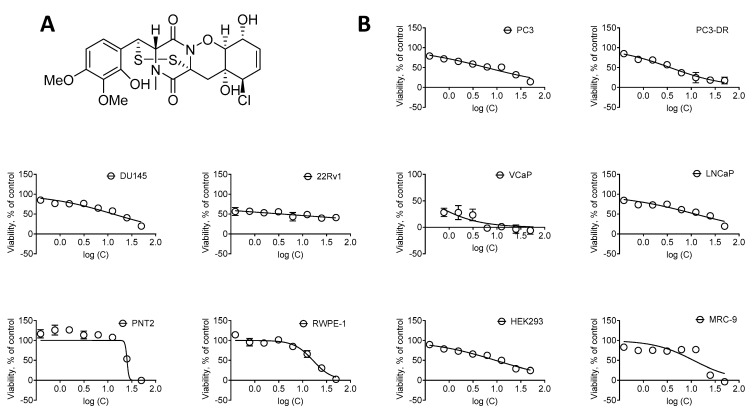
Cytotoxic activity of NB. (**A**) The structure of NB. (**B**) Cytotoxicity of NB in human prostate cancer cell lines versus human noncancer cell lines following 48 h of treatment. Cell viability was determined using MTT assay. Number of replicates *n* = 3. The calculated IC_50_s are represented in Table 1.

Remarkably, halogenated analogs of glioverin were reported to be more bioactive in comparison to nonhalogenated molecules [13,14]. N-methylpretrichodermamide B (NB) is a glioverin derivative containing chlorine and a disulfide bridge in its structure (Figure 1A). Initially, this natural compound was isolated from the fungus *Penicillium* sp. found in an Egyptian hypersaline lake [8]. Simultaneously, NB was isolated from a sponge-derived *Penicillium adametzioides* strain by the group of Huang and Wang, who described it as a new adametizine A [11]. More recently, NB was reisolated by us from an ethyl acetate extract of the algae-derived fungus *Penicillium* sp. KMM 4672 [9]. In comparison with nonchlorinated analogs, NB exhibited more pronounced activity against *Artemia salina* (LD_50_ 4.8 µM) and antimicrobial activity in various pathogenic bacteria (MIC 8–32 µm/mL) [11]. Notably, in this study, no significant anticancer activity in a panel of 14 human cancer cell lines, including human lung, cervical, liver, breast, gastric, pancreatic, colon, and glioma cancer cells, and one prostate cancer cell line DU145, was reported (IC_50_ > 10 µM) [11]. Furthermore, the other group reported a significant cytotoxicity against mouse lymphoma cells L5178Y with IC_50_ = 2 µM [8]. Moreover, recently, we also described a pronounced cytotoxic activity of this marine natural compound in human prostate cancer 22Rv1, PC-3, and LNCaP cells with IC_50_ = 0.51, 5.11, and 1.76 µM, respectively [9]. Interestingly, NB was found to be active in hormone-independent (and therefore, drug-resistant) 22Rv1 cells at submicromolar concentrations, while no hemolytic activity (IC_50_ ≥ 100 µM) and only minor cytotoxic activity in splenocytes (ID_50_ = 62.1 µM) was observed [9]. Despite the promising activity, no data on the mechanism of action in mammalian cells are available to date.

Interestingly, for the other natural compounds gliotoxin, chaetocin, and chetomin, which also belong to the family of epithiodiketopiperazines, a promising in vitro and in vivo activity has been reported in human prostate cancer cell models [15,16]. Considering our recent study concerning the potent activity of NB in prostate cancer cells [9], we hypothesized that NB may have a potential as a lead compound for prostate cancer therapy. Therefore, we aimed to further explore the activity, selectivity, and the mode of action of NB in this cancer entity using functional kinome analysis, among others.

Here, we report the anticancer activity of NB in an extended panel of human prostate cancer cells alone and in combination with established drugs already used against prostate cancer. In addition, we report, for the first time, relevant aspects of the mechanism of biological activity and an effect on nonmalignant cells.

## 2. Results and Discussion

### 2.1. Cytotoxicity and Selectivity of NB in Human Prostate Cancer Cells

To examine the anticancer potential and selectivity of NB, we used a panel of six human prostate cancer cell lines, harboring different levels of treatment resistance to representatively reflect the heterogeneity of the disease. This panel included: (i) androgen receptor (AR)-negative DU145 and PC3 cells exhibiting a resistance to various hormonal and standard chemotherapeutics [17]; (ii) docetaxel-resistant PC3-DR cells derived from PC3 cells by a long-term exposure to docetaxel and showing ~50-fold less sensitivity to this drug compared to their parental cell line [18]; (iii) AR-FL- (AR full length) and AR-V7-positive (AR splice variant V7) hormone-resistant 22Rv1 and VCaP cells [17,19]; (iv) AR-FL-positive hormone-sensitive LNCaP cells. Additionally, four human noncancer cells lines were utilized in order to determine the selectivity of NB towards prostate cancer cells, i.e., prostate noncancer PNT2 and RWPE-1 cells, human embryonic kidney HEK 293T cells, and human fibroblasts MRC-9. The different cell lines were treated with NB and the cell viability was examined using MTT assay (Figure 1). The mean IC_50_ of NB and docetaxel (used as a reference substance) in noncancerous cell lines was divided by the mean IC_50_ in the prostate cancer cell lines to determine the selectivity index (SI). NB was found to be selective towards prostate cancer cells as indicated by a SI of 2.4 (Table 1).

In line with the previous report by the group of Huang and Wang [11], the IC_50_ of NB detected by us in DU145 was 12.97 ± 2.69 µM (IC_50_ > 10 µM) (Table 1). Furthermore, NB was found to be the most cytotoxic in 22Rv1 and VCaP cancer cells, while displaying cytotoxicity at micromolar concentrations in all cell lines investigated (Table 1). Remarkably, the IC_50_ of NB in docetaxel-resistant PC3-DR cells was twofold lower compared to the parental PC3 cells. This suggests lack of cross-resistance between NB and docetaxel (Figure 1, Table 1).

**Figure 2:**
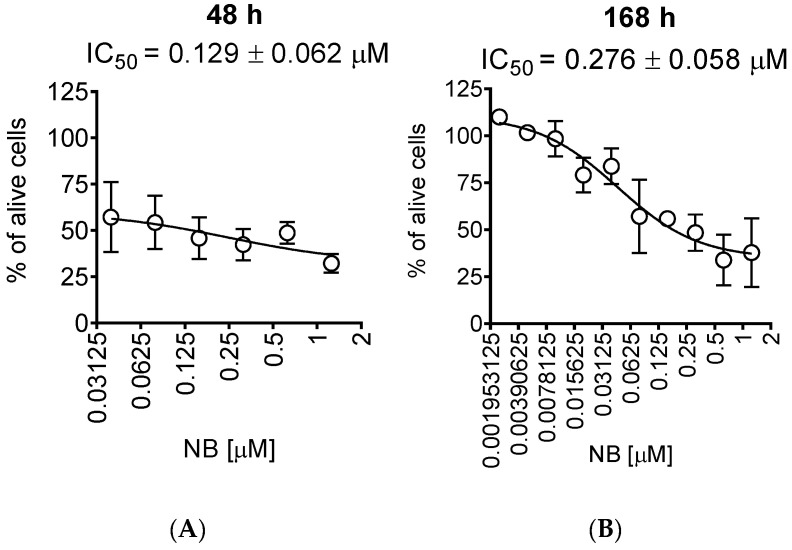
Trypan blue exclusion assay. 22Rv1 cells were treated for 48 h (**A**) or 168 h (**B**) with the indicated concentrations of NB. The cells were harvested, stained, and counted with an automatic Vi-CELL cell counter device. Number of replicates *n* = 3.

For further investigations, we selected human prostate cancer 22Rv1 cells, since they displayed one of the highest sensitivities to NB. Moreover, 22Rv1 cells are known to express not only AR-FL but also AR-V7: an AR splice variant responsible for resistance to AR-targeted therapy. Thus, these cells represent an aggressive hormone-independent prostate cancer subgroup. The MTT assay assesses the metabolic activity of the cells [20] by determining the mitochondrial reducing capability. Trypan blue exclusion assay, on the other hand, differentiates between the cells with intact (trypan blue negative alive cells) or disrupted (trypan blue positive dead cells) cellular membrane and is usually used for the assessment of cellular proliferation [21]. Interestingly, the cytotoxic effect of NB determined by trypan blue exclusion assay was more pronounced than the effect determined using MTT assay (Figure 2, Table 1). In both MTT and trypan blue exclusion assays, a statistically significant suppression of cellular viability was already observed at concentrations much lower than IC_50_s (Figure 1B and Figure 2A). Therefore, we speculated that a longer treatment time may result in a further IC_50_ decrease and, consequently, we assessed the cytotoxic activity of NB after 7 days of treatment (Figure 2B). However, the activities determined 48 h and 168 h following the treatment were comparable (Figure 2A,B). This finding suggests that 48 h is an optimal time for the investigation of the anticancer effects of NB.

**Figure 3:**
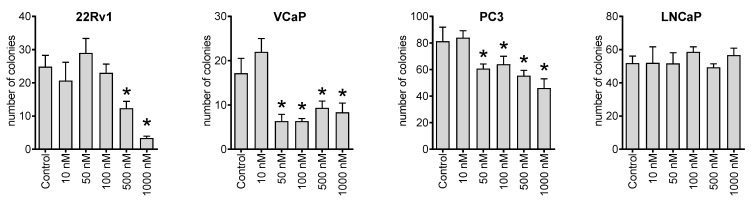
Colony formation assay. The human cancer cells were seeded in 6-well plates and treated for 48 h with the indicated concentrations of NB. Then, the media was exchanged, and the plates were incubated for 14 days, which was followed by fixation, staining, and counting. Number of replicates *n* = 3. Statistical significance: * *p* < 0.05 (ANOVA).

Next, we investigated the effect of NB on the ability of cancer cells to form colonies from single cells using a colony formation assay and a preplating mode. Thus, we observed that the compound could inhibit the colony-formation ability of 22Rv1, VCaP, and PC3 cell lines at nanomolar concentrations (Figure 3). This suggests a possible antimetastatic activity reflecting a suppression of metastases formation from single cancer cells. However, this speculation awaits further confirmation in vivo.

**Figure 4:**
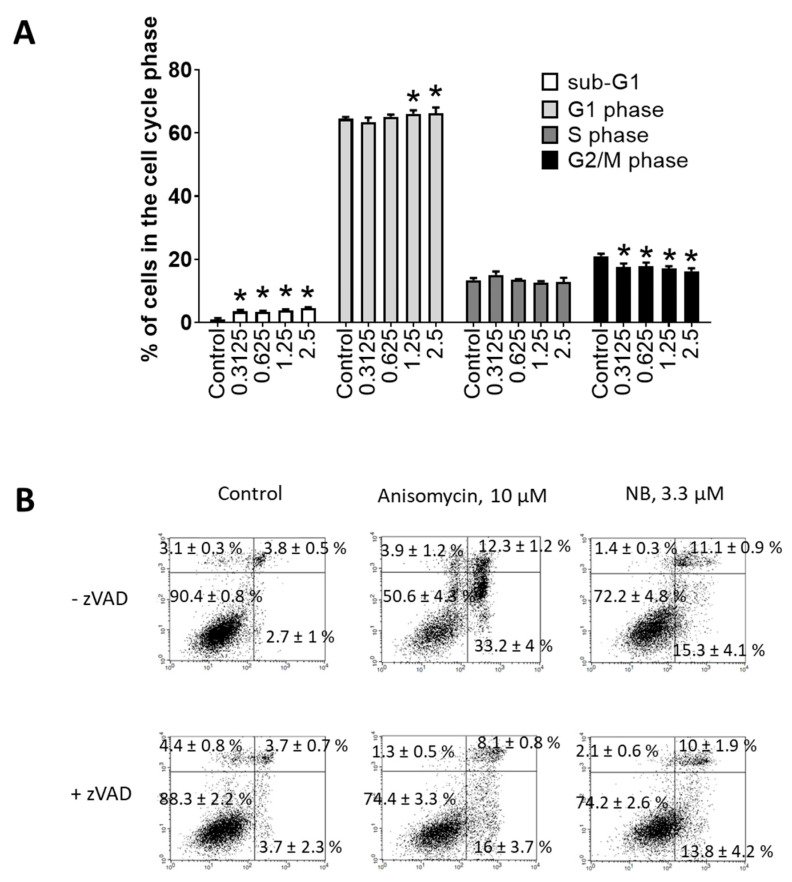
Investigation of the proapoptotic activity of NB. Flow cytometry analysis of 22Rv1 cells after 48 h of treatment. (**A**) Cell cycle and DNA fragmentation analysis. Treated cells were stained with PI and analyzed using a flow cytometry technique. Sub-G1 population was assumed to be apoptotic cells containing fragmentated DNA. (**B**) Analysis of phosphatidylserine externalization. Treated cells were double-stained with annexin-V-FITC and PI and analyzed using the flow cytometry technique. Cells of the low left quadrant (annexin-V-FITC^−^/PI^−^) were considered as alive cells. The Cell Quest Pro v.5.2.1 software (BD Bioscience, San Jose, CA, USA) was used to analyze and quantify the FACS data. Cells treated for 48 h with 10 µM of anisomycin were used as a positive control. Number of replicates *n* = 3. Statistical significance: * *p* < 0.05 (ANOVA).

Flow cytometry was further used to investigate potential apoptosis-inducing effects of NB. Consequently, dose-dependent DNA fragmentation (Figure 4A) and phosphatidylserine externalization (Figure 4B) were examined. Anisomycin—a well-established inductor of caspase-dependent apoptosis—was used as a positive control. NB treatment caused DNA fragmentation and phosphatidylserine externalization in 22Rv1 cancer cells, suggesting apoptotic cell death. To obtain further insights into apoptosis induction by NB, we tested the role of caspases in drug-induced cell death. The cells were pretreated with pan-caspase inhibitor z-VAD(OMe)-fmk (zVAD) and then co-treated with NB or anisomycin. However, pretreatment with zVAD could not significantly inhibit NB-induced death of 22Rv1 cells, while the cytotoxic effect of anisomycin was strongly inhibited (Figure 4B). These results suggest that biological processes different from the classical caspase-dependent apoptosis are involved in the anticancer effect of the isolated natural compound. This finding suggests NB to also be active in cancer cells bearing mutated or inactive caspase genes, which would normally be resistant to classical apoptosis-inducing agents [22]. Additionally, a small but significant effect on cell cycle progression was detected in the treated 22Rv1 cells (Figure 4A). In previous studies, the halogenated glioverin derivatives exhibited a higher cytotoxic activity in comparison to nonhalogenated molecules [13,14]. Thus, trichodermamide B—a modified dipeptide chlorinated analog of glioverin—exhibited high cytotoxic activity in HeLa cells, while nonchlorinated trichodermamides A and C were inactive [14]. In this study, the mechanism of action of trichodermamide B was identified as DNA damage, which leads to the activation of Chk1/2, S-phase cell cycle arrest and ultimately to cancer cell apoptosis [14]. Interestingly, other synthetic derivatives, which in contrast to trechodermamide B did not possess the C-4/C-5 chlorohydrin moiety, were also cytotoxic; however, they had a different mode of action, which lacks DNA-damaging activity [14]. In line with the previous study, we showed that NB also induced DNA damage, which was indicated as DNA fragmentation and led to cell cycle arrest. Although in 22Rv1 cells a G1-phase arrest was observed, it is known that DNA damage and Chk1/2 activation may result in either G1, S-, or G2/M-phase cell cycle arrest, depending on the model used and the nature of the stimulus [23]. Thus, our data support previous speculations on the DNA-damaging activity of trichodermamide B and related compounds containing the chlorine moiety.

In contrast to trichodermamide B, apart from the chlorine moiety, NB also contains a disulfide bridge in its structure (Figure 1A). Gliotoxin—the very first member of the epithiodiketopiperazines family—also contains a disulfide bridge and its mode of action has been studied in more detail. Gliotoxin was reported to execute its cytotoxic action via three main mechanisms, specifically inhibition of proteosomes [24], generation of reactive oxygen species (ROS), and covalent binding to protein thiol groups [25,26]. Generation of ROS is a well-established factor that leads to DNA damage, while the presence of a disulfide bridge is responsible for binding to protein thiol groups and proteosome inhibition [27]. It is likely that NB exerts similar effects, which ultimately result in the observed cytotoxic effects. This speculation is to be examined in the future.

Overall, NB demonstrates a promising anticancer activity in vitro that can partially be explained by the induction of cellular death, which has an underlying mechanism different from classical caspase-dependent apoptosis. It is important to note that further in vivo pharmacokinetics and pharmacodynamics studies are required to validate the anticancer potential of NB in prostate cancer. Previously, an in vivo activity in a human prostate cancer model was reported for another three natural epithiodiketopiperazines, namely, gliotoxin, chaetocin, and chetomin [15]. Therefore, it is likely that NB is also effective in vivo; however, this speculation requires experimental validation.

### 2.2. NB Affects Protein Tyrosine Kinases’ Activity

**Figure 5:**
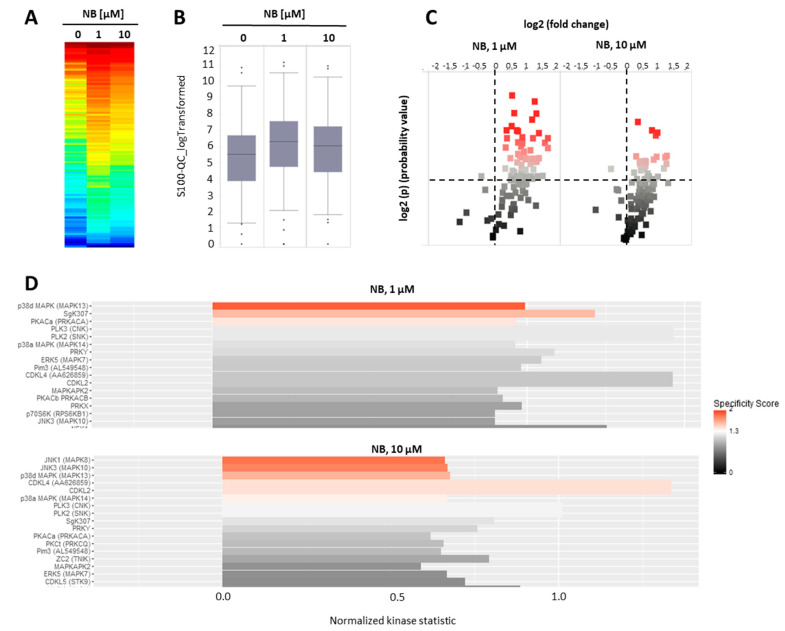
Kinome profiling of serine/threonine kinases (STKs). 22Rv1 cells were incubated with NB for 2 h; the proteins were extracted and STK were analyzed using functional kinome profiling. The heatmap displays the log2-transformed signal intensities for each sample. The signals were sorted from high (red) to low (blue) intensity/phosphorylation (**A**). The overall peptide phosphorylation and, with that, the overall kinase activity is shown by the box plot (**B**). The volcano-plot identifies significantly altered peptides. Red dots indicate significantly increased phosphorylation of peptide substrates compared to control samples (log_2_(*p*) > 1.3, dotted line (**C**). (**D**) Upstream kinase analysis indicating effect of the treatment on STK in 22Rv1. The top kinases predicted to be affected are shown, with all kinases displaying normalized kinase statistic > 0 (*X*-axis), meaning higher activity of these kinases in cells treated with NB in comparison to control; specificity score > 1.3 indicates statistically significant changes. Number of replicates *n* = 3.

To further investigate the mechanism of anticancer action of NB, we examined the effects of the compound on serine-/threonine protein kinases. Protein kinases modify target molecules (proteins) via phosphorylation, thus, altering their activity. A subgroup of these kinases—serine-/threonine kinases (STK)—play a key role in various essential processes related to cell death and cell survival [28]. Consequently, protein kinases, in general, are important target molecules for various anticancer drugs [29]. Specifically, modification of STKs’ activity has been exploited as a mechanism of action. Several clinically approved drugs, such as sunitinib, palbociclib, cobimetinib, and axitinib, utilize this mechanism to execute their therapeutic action [28]. Thus, we performed functional kinomics using the microarray-based PamGene technology. This method allows a systematic examination of the changes in STK activity (http://www.pamgene.de, accessed on 22 January 2022, Figure 5A–D) [30]. We used two different concentrations, specifically, 1 µM and 10 µM, in order to cover different biological effects, which may take place at the different drug doses (Figure 5A). To reduce the detection of unspecific effects that are related to or caused by cell death, a short treatment time was chosen. After 2 h, we detected an overall increase in peptide phosphorylation and kinase activity in the treated samples, respectively (Figure 5A,B), with several peptides displaying significantly increased phosphorylation in the treated group (Figure 5C). The upstream kinase analysis identified JNK1/2 and p38 kinases to be very likely upregulated in the treated samples (Figure 5D), both belonging to the mitogen-activated protein kinases (MAPKs). MAPKs are involved in many different cancer-related processes, such as cell survival, but also cell death. For prostate cancer, MAPKs have been demonstrated to be strongly associated with tumor growth [31,32,33]. Furthermore, AR-signaling and, in particular, JNK1/2 were reported to influence each other through cross-talk [34,35,36]. Because of the ambiguous impact on cancer cell growth and elimination, we investigated the effect of NB on MAPKs in depth [29,32].

**Figure 6:**
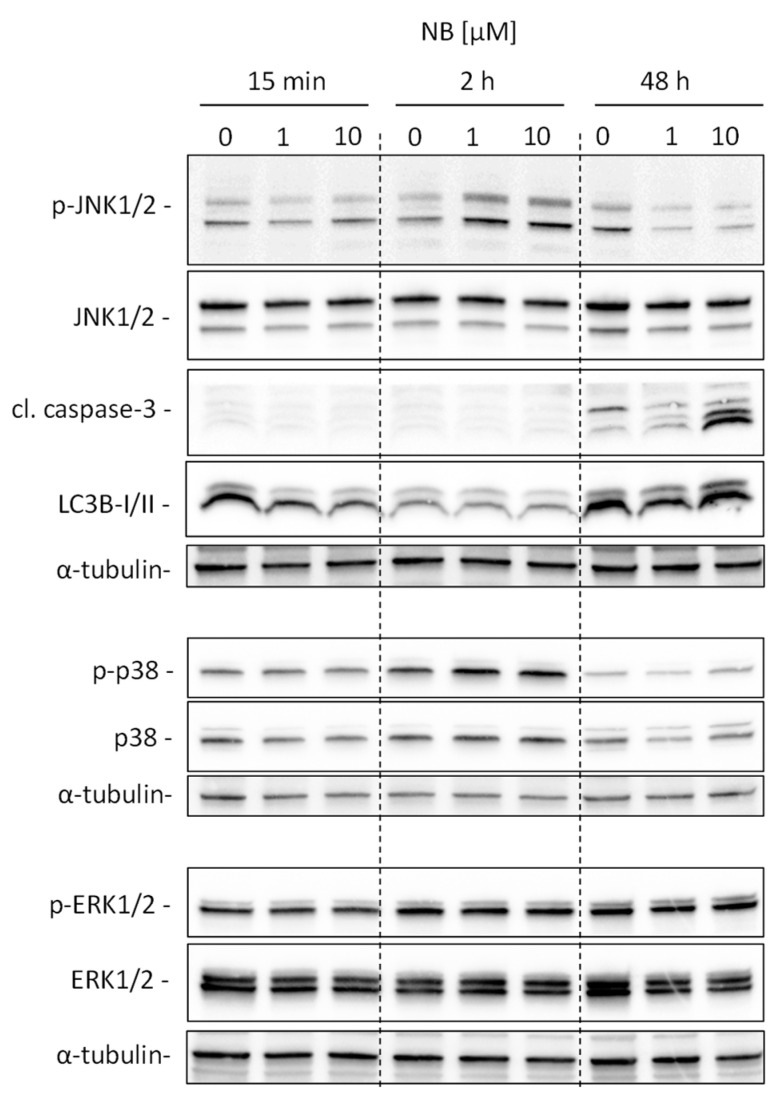
Validation of kinome analysis data. 22Rv1 cells were treated with 1 µM or 10 µM of NB for the indicated time. The protein expression was analyzed by Western blotting using total protein load equal to 30 µg/slot. α-Tubulin was used as a loading control.

Our analysis suggests kinases JNK1 and p38delta to be the most likely activated in NB-treated prostate cancer cells (Figure 5D). To validate and further classify these results, we performed Western blotting analyses of JNK1/2 and p38 MAPK activation. We examined several time points, specifically, 15 min, 2 h, and 48 h (Figure 6). A pronounced JNK1/2 phosphorylation and activation of p38 were detected after 2 h of treatment with NB, while no activation of other MAPK ERK1/2 (which was not predicted to be significantly affected under the treatment) was detected (Figure 6). Of note, no phosphorylation (or even slight downregulation) of JNK1/2 was found after 48 h of exposition to NB (Figure 6). The latter effect may be a result of cell death-related events, which was suggested by the caspase-3 cleavage that was observed 48 h after drug exposure. Notably, no significant effect on autophagy was detected as suggested by the lack of alteration of LC3B-I/II expression in the treated cells (Figure 6). Hence, our results indicate JNK1/2 and p38 phosphorylation to be one of the first drug-induced cellular events when exposed to NB (2 h time point, Figure 6), before any signs of cytotoxicity can be detected (48 h time point, Figure 6).

### 2.3. Effect of JNK1/2 and p38 MAPKs on the Cytotoxicity of NB

**Figure 7:**
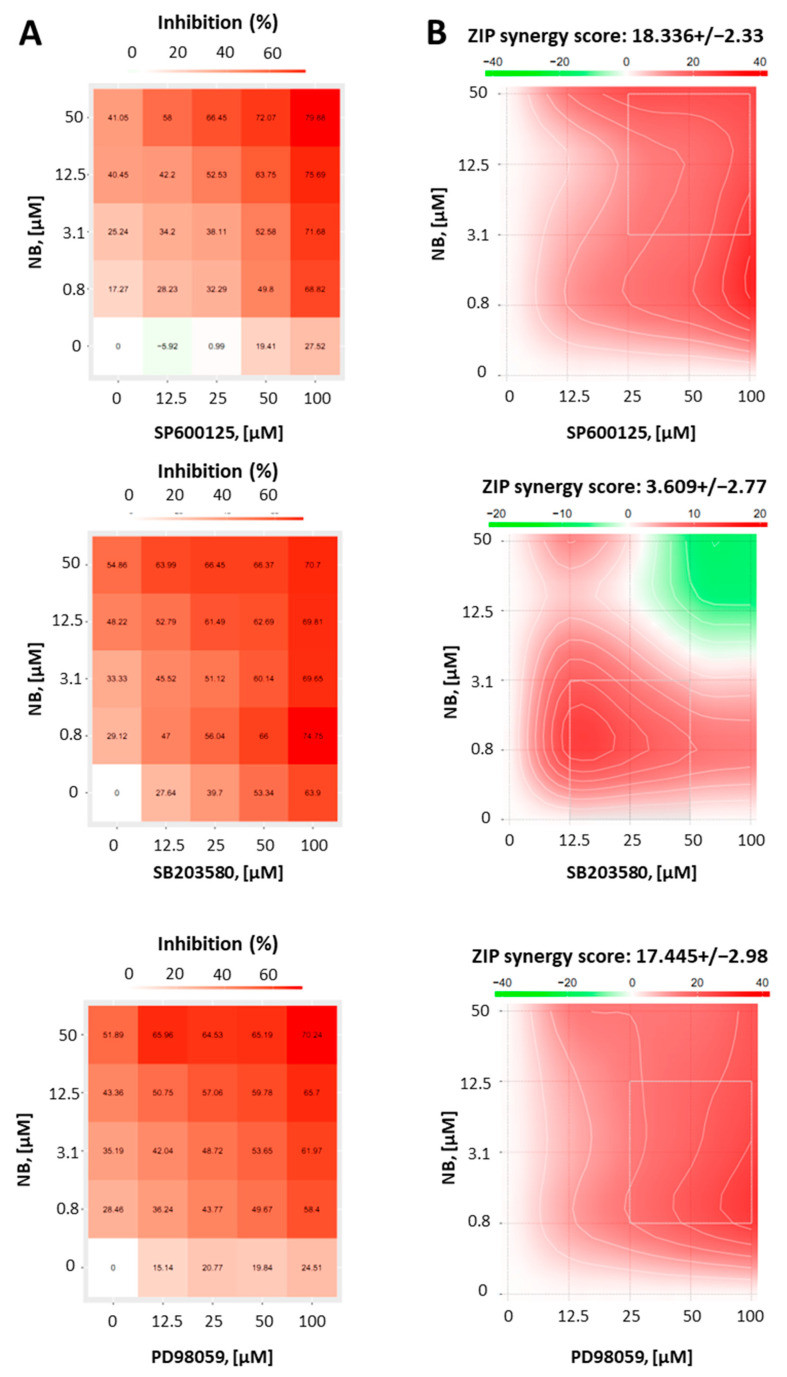
Results of combinational treatment of NB and different MAPK-inhibitors. 22Rv1 cells were pretreated with JNK1/2 inhibitor SP600125, p38 inhibitor SB203580, or MEK1/2 inhibitor PD98059 at the indicated concentrations for 1 h, followed by treatment with the indicated concentrations of NB for 24 h (number of replicated *n* = 3). The MTT assay was used to evaluate the viability of the treated cells (**A**). To calculate and visualize the effect of the drug combination (synergism/additive effect/antagonism), the SynergyFinder 2.0 software and a ZIP reference model were used (**B**). Green regions indicate antagonism, white regions refer to additive effect, and red regions indicate synergism.

Whether activation of MAPKs contribute to cell death or survival depends not only on the cellular context, but also on the nature of the activating stimuli [37]. Thus, to reveal the function of JNK1/2 and p38 in the anticancer effect of NB, we co-treated 22Rv1 cells with NB and specific inhibitors of the above-mentioned MAPKs. Therefore, selective JNK1/2 inhibitor SP600125, p38 inhibitor SB203580, and MEK1 inhibitor PD98039 (Figure 7) were used. As MEK1/2 kinase directly and exclusively activates ERK1/2, PD98039 inhibitor is often used as an ERK1/2 inhibitor [38]. Thus, an inactivation of MEK1/2 leads solely to an inhibition of ERK1/2 [38]. Since the inhibitors themselves exert cytotoxic effects in cancer cells, we used a Chou–Talalay method to distinguish between synergism, additive effects, or antagonistic effects of the particular MAPK inhibitor on the compound bioactivity. For this, using an MTT assay, we determined a cytotoxic effect on various concentrations of NB, MAPK inhibitors, and their combinations in 22Rv1 cells. The viability data were further analyzed using the SynergyFinder v. 2.0 software (Network Pharmacology for Precision Medicine in the Research Program of System Oncology, Faculty of Medicine at University of Helsinki, Helsinki, Finland) and a Zero interaction potency (ZIP) reference model [39] (Figure 7). Hence, the heatmaps displaying the percentage of dead cells in each treatment setting were generated (Figure 7A). Remarkably, our investigations suggest a pronounced synergism of NB in combination with JNK1/2-inhibitor SP600125 and p38-inhibitor SB203580, indicating the activation of both kinases in response to NB treatment is a prosurvival mechanism, which reduces NB cytotoxicity (Figure 7B). In addition, the MEK1/2-inhibitor PD98039 displayed a similar effect when combined with NB (Figure 7B). Thus, an active MEK/ERK pathway also seems to be important for cellular survival in this particular context and to help the cancer cells to overcome the cytotoxic effect of NB.

In conclusion, NB treatment primarily induces JNK1/2 and p38 kinases, suggesting the activation of prosurvival stimuli in the prostate cancer cells. The combination with correspondent JNK1/2- and p38-inhibitors resulted in synergistic effects with increased cytotoxic effects of NB. Of note, while our kinome profiling analysis did not display any significant changes in ERK1/2 activity, the inhibition of the MEK/ERK pathway in NB-treated cells revealed synergistic effects, indicating its involvement in survival mechanisms, similar to the JNK1/2 and p38 pathways (Figure 7B). Therefore, a combination with MAPK inhibitors should be considered as a strategy in further preclinical and possible clinical trials.

### 2.4. Inhibitory Effect of NB on P-Glycoprotein Activity and Synergism with Docetaxel

**Figure 8:**
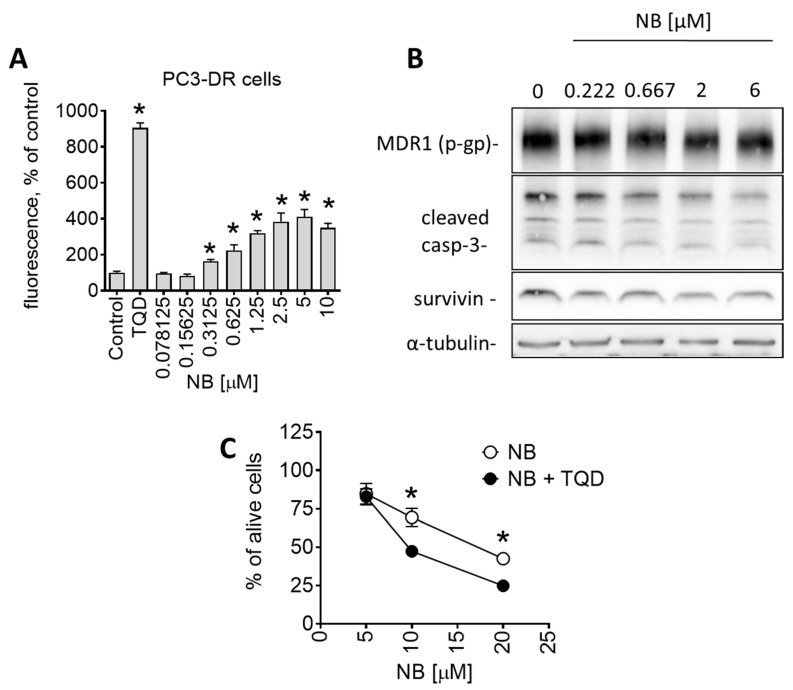
Effect of NB on p-gp expression and activity of PC3-DR cells. (**A**) Effect of NB on calcein accumulation (measures as green fluorescence) in PC3-DR cells. The effect was measures following 30 min incubation with the drug. P-gp inhibitor tariquidar (TQD, 50 nM) was used as a positive control. Number of replicates *n* = 3. (**B**) Western blotting analysis of protein expression in PC3-DR cells after 48 h of treatment with the indicated concentrations of NB. (**C**) Effect of NB on the viability of PC3-DR cells. Cells were treated with NB for 48 h with or without 30 min pretreatment with tariquidar (TQD, 50 nM) for 30 min. Viability was measured using an MTT assay. Number of replicates *n* = 3. Statistical significance: * *p* < 0.05 in ANOVA (**A**) or Student’s *t*-test (**C**).

Another interesting observation was the pronounced sensitivity of drug-resistant PC3-DR to NB (Table 1). PC3-DR cells were generated via continuous treatment of parental PC3 cells with stepwise increasing nonlethal doses of docetaxel [18]. They are ~50-fold more resistant to docetaxel compared to the parental cell line PC3 [40]. Interestingly, NB displayed a higher cytotoxicity level in PC3-DR in comparison to docetaxel-sensitive PC3 (Table 1). It is well established that a main factor mediating resistance of PC3-DR cells to docetaxel is the overexpression of p-glycoprotein (p-gp, MDR1) [41]. P-gp is a transmembrane pump protein that mediates the efflux of various drugs, including docetaxel, out of cancer cells, which results in resistance to chemotherapeutic agents. Indeed, we have recently reported p-gp overexpression in PC3-DR cells, which explains the measurable resistance to docetaxel [42]. Since there was no cross-resistance in PC3-DR cells to NB (Table 1), we speculated that the tested compound is not excreted by the efflux pump. To further understand the interactions of NB and p-gp, and the effect of NB on p-gp activity, we performed a calcein-AM-exclusion assay. Consequently, p-gp overexpressing PC3-DR cells were chosen as a test model. This assay utilizes calcein-AM (calcein acetoxymethyl ester), a nonfluorescent dye that enters cells through passive diffusion and exits cells through p-gp. Metabolic active cells contain cytosolic esterases that hydrolyze calcein-AM into its green-fluorescent form (calcein). The green fluorescence can be further measured. However, in p-gp overexpressing cells, calcein-AM is excreted out of the cell before it is metabolized. As a result, these cells do not accumulate calcein. Application of p-gp inhibitors, such as tariquidar (TQD), leads to the accumulation of calcein-AM/calcein inside the cells and, therefore, to increased fluorescence. Hence, calcein-AM can be used to monitor p-gp activity and allows for the examination of small molecules as potential p-gp inhibitors. In our experiments, PC3-DR cells were exposed to NB for 30 min followed by incubation with calcein-AM. Remarkably, NB treatment of PC3-DR cells had already significantly increased the green fluorescence of the cells at nanomolar concentrations in a dose-dependent manner, suggesting treatment-induced inhibition of p-gp (Figure 8A).

It is important to note that a reduction in p-gp activity could be conditioned by p-gp degradation or cell death-related processes. Consequently, using a Western blotting analysis, we measured p-gp expressional levels in the treated PC3-DR cells. We detected no significant changes in p-gp expression, even at high concentration of 6 µM (Figure 8B). Thus, degradation of p-gp does not appear to be the reason for the observed reduced p-gp activity. Similarly, a cleavage of caspase-3 in PC3-DR was only detected at high concentrations of 6 µM (Figure 8B), while an inhibitory effect on p-gp activity was already detected at the noncytotoxic concentration of 0.3125 µM (Figure 8A). Thus, the observed effect on p-gp activity seems to be exclusively related to the interaction of NB with p-gp rather than result from any cell death-related events.

As a next step, we investigated how NB interacts with p-gp. P-gp activity can be reduced in two distinct ways: (i) competitive inhibition through direct binding to the active pocket as the ligand itself; and (ii) allosteric/noncompetitive inhibition reducing the activity of a protein through binding to allosteric/regulatory sites or other means. For example, docetaxel and some other taxanes are not only p-gp substrates but can also inhibit efflux of calcein and other drugs via competition for p-gp binding. Therefore, docetaxel binds to p-gp (while being excreted from the cell), competitively inhibiting p-gp-mediated calcein-AM excretion, which leads to calcein accumulation, resulting in more intense green fluoresce. To elucidate the mechanism of p-gp inhibition by NB (competitive vs. allosteric/noncompetitive inhibition), we co-treated PC3-DR cells with tariquidar (TQD), a selective noncompetitive p-gp inhibitor and NB, and examined the effect on cellular viability (Figure 8C) [43]. Recently we showed an increase in docetaxel’s cytotoxic activity in PC3-DR cells when combined with TQD [42]. The taxane accumulates inside cancer cells due to TQD-induced p-gp blockade, resulting in decreased docetaxel excretion, resulting in a higher cytotoxic effect [44]. Importantly, the cytotoxicity of NB was also increased when co-treated with TQD, indicating excretion of NB via p-gp, similar to docetaxel (Figure 8C).

Based on these results, we hypothesized that NB is a competitive inhibitor of p-gp, which, at the same time, has certain substrate-like features and, similar to taxanes, can be partially excreted from the cancer cell via the p-gp system. Although the efficacy of p-gp inhibition by NB was rather high, the cytotoxicity increase when combined with TQD was not as pronounced (Figure 8C) as it was for docetaxel (recently reported in [42]). Interestingly, a certain degree of p-gp-mediated excretion of NB due to its substrate-like activity, unlike with docetaxel, did not result in a lower cytotoxicity of NB in PC3-DR cells. In contrast, NB was more active in PC3-DR cells when compared to PC3 cells (Table 1). Therefore, it is likely that other effects, which are still to be discovered, are involved in the anticancer activity of NB. Thus, NB holds promising potential as a potent p-gp inhibitor and a promising candidate for treatment of drug-resistant prostate cancer. Moreover, it should be noted that, as a p-gp inhibitor, NB was not as potent as TQD (Figure 8A), probably due to the low affinity to this transport protein. Therefore, further investigation of the NB biological effects and structural optimization may be useful to increase its p-gp-inhibitory properties and decrease its substrate-like features. Apart from tumor cells, p-gp is expressed at a detectable level in the intestine, placenta, kidney, liver, and brain, and in some other noncancer tissues [45]. Therefore, toxicity of NB in these organs should be carefully examined.

**Figure 9:**
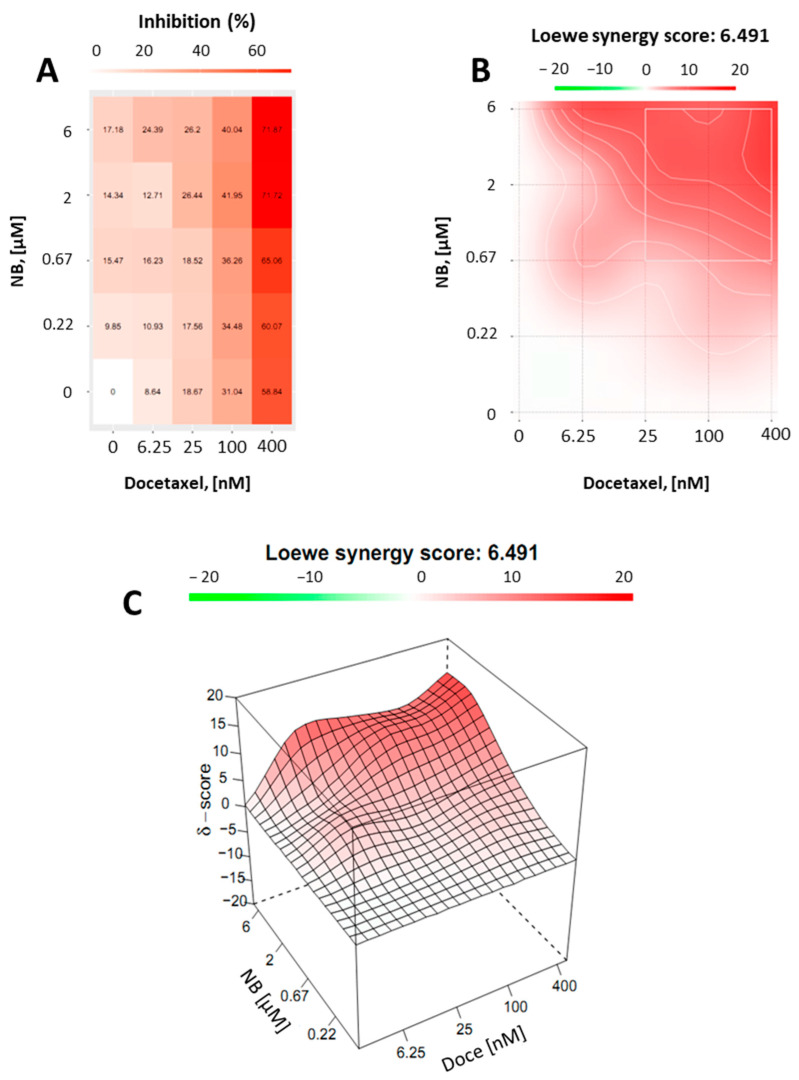
Combinational treatment on PC3-DR cells with NB and docetaxel. The cells were co-treated with indicated concentrations of NB, docetaxel, or their combination for 24 h. MTT assay was used to examine cellular viability. Number of replicates *n* = 3. (**A**) The heatmap represents cytotoxicity (viability inhibition) of the individual drugs or their combinations. (**B**,**C**) To calculate and visualize in 2D (**B**) or 3D (**C**) the effect of the drug combination (synergism/additive effect/antagonism), the SynergyFinder v. 2.0 software (Network Pharmacology for Precision Medicine in the Research Program of System Oncology, Faculty of Medicine at University of Helsinki, Helsinki, Finland) and a ZIP reference model were used. Green regions indicate antagonism, white regions refer to additive effect, and red regions indicate synergism.

Finally, we assumed that, due to the p-gp-inhibitory activity, similar to TQD, NB may be capable of enhancing docetaxel cytotoxicity in PC3-DR cells. Thus, drug-resistant PC3-DR cells were treated with docetaxel in combination with NB (Figure 9). Potential synergism between NB and docetaxel was measured by the Chou–Talalay method as described before. Indeed, we found a synergistic effect of both drugs when applied in combination (Figure 9). This finding was in line with previous results, further validating our hypothesis regarding the competitive inhibition of p-gp by NB.

## 3. Materials and Methods

### 3.1. Isolation of NB

N-methylpretrichodermamide B (NB) was isolated from EtOAc extract of *Penicillium* sp. KMM 4672 (brown alga *Padina* sp., South China Sea, Vietnam), using column chromatography on various sorbents followed by purification using normal- and reverse-phase HPLC as was previously described [9]. The purity of the compound was established using NMR, HPLC, and mass spectrometry data [9].

### 3.2. Reagents and Antibodies

The following reagents and antibodies were used for biological experiments: anisomycin (NeoCorp, Weilheim, Germany); annexin-V-FITC (BD Bioscience, San Jose, CA, USA); docetaxel (Pharmacy of the University Hospital Hamburg-Eppendorf, Hamburg, Germany); SP600125 (JNK1/2 inhibitor) and SB203580 (p38 inhibitor) (LC Laboratories, Woburn, MA, USA); PD98059 (MEK inhibitor) (Merck Chemicals GmbH, Darmstadt, Germany); PhosphoSTOP™ *EASY*packs phosphotase inhibitors cocktail and cOmplete™ *EASY*packs protease inhibitors cocktail (Roche, Mannheim, Germany); propidium iodide (PI), MTT (3-(4,5-dimethylthiazol-2-yl)-2,5-diphenyltetrazolium bromide) (Sigma, Taufkirchen, Germany); RNase (Carl Roth, Karlsruhe, Germany); tariquidar (p-glycoprotein inhibitor) (MedChemExpress, Monmouth Junction, NJ, USA); primary and secondary antibodies used are listed in Table 2.

### 3.3. Cell Lines and Culture Conditions

All cell lines used for the experiments were recently authenticated by a commercial service (Multiplexion GmbH, Heidelberg, Germany). The cultured cells had a passage number < 30. The following cell lines were purchased from ATCC (Manassas, VA, USA): human prostate cancer cell lines PC-3, DU145, 22Rv1, and LNCaP, and human prostate noncancer cell line PNT2; the following cell lines were purchased from ECACC (Salisbury, UK): HEK 293T (human embryonic kidney cells) and MRC-9 (human fibroblast cells). Docetaxel-resistant human prostate cancer cells PC3-DR were generated by long-term cultivation of PC3 cells, in increasing concentrations of docetaxel and were kindly provided by Su Jung Oh-Hohenhorst and Prof. Z. Culig [18].

The following culture conditions were applied: cells were cultured as monolayers at 37 °C in a humidified atmosphere with 5% (*v*/*v*) CO_2_ in the correspondent culture medium. The medium used for PNT2, LNCaP, 22Rv1, PC3, and DU145 cells was 10% FBS/RPMI medium (RPMI medium supplemented with Glutamax^TM^-I (gibco^®^ Life technologies^TM^, Paisley, UK) containing 10% fetal bovine serum (FBS, gibco^®^ Life technologies^TM^) and 1% penicillin/streptomycin (Invitrogen)); for MRC-9 and HEK 293 cells, it was 10% FBS/DMEM medium (DMEM medium supplemented with Glutamax^TM^-I (gibco^®^ Life technologies^TM^) containing 10% FBS and 1% penicillin/streptomycin (gibco^®^ Life technologies^TM^)). PC3-DR cells were cultured in 10% FBS/RPMI medium additionally containing 12.5 nM of docetaxel. Cells were regularly checked for mycoplasma infection and stable phenotype and were kept in culture for a maximum of 3 months.

### 3.4. MTT Assay

The MTT assay was used to monitor the effect of the drugs on cell viability in terms of metabolic activity, as previously reported [46]. First, preincubation of the cells in 96-well plates (6 × 10^3^ cells/well in 100 μL/well) was performed overnight in the correspondent culture medium. Next, the medium was exchanged for fresh medium containing the investigated compounds or a vehicle (100 μL/well). Cells were then incubated for 48 h, unless otherwise stated. Then, 10 μL/well of 3-(4,5-dimethylthiazol-2-yl)-2,5-diphenyltetrazolium bromide reagent (MTT, 5 mg/mL) were added. After 2–4 h of additional incubation the media was removed, and the plates were dried. Finally, 50 μL/well of DMSO were added to solve the formed formazan crystals produced by the metabolic active cells and Infinite F200PRO reader (TECAN, Männedorf, Switzerland) was used to measure the cell viability. The results were proceeded with a GraphPad Prism software v.9.1.1 (GraphPad Software, San Diego, CA, USA). Cells treated with the solvent of the drugs alone were used as a control (100% alive cells). The effect of the drugs on cell viability is represented as IC_50_.

### 3.5. In Vitro Trypan Blue Exclusion Assay

The trypan blue exclusion assay was used to monitor the effect of the drugs on cell viability regarding cellular membrane integrity and was performed as described before [46]. In brief, cells (2 × 10^5^ cells/well) were seeded in 6-well plates (1 mL/well) and incubated overnight. Next, the media was substituted with fresh media (1 mL/well) containing drugs at indicated concentrations. Cells were treated for 48 h, harvested by trypsination, and stained with trypan blue. The automatic Beckman Coulter Vi-CELL (Beckman Coulter, Krefeld, Germany) was used to count trypan blue-positive and -negative cells. Trypan blue-negative cells were postulated to have an intact cellular membrane (i.e., alive cells), whereas trypan blue-positive cells were assumed to have damaged cellular membrane (i.e., dead cells).

### 3.6. Profiling of Serine-/Threonine Kinase Activity

Profiling of kinase activity was executed as described before [47,48]. Briefly summarized, the affected serine-/threonine kinases were profiled using a PamStation^®^12 (PamGene International, ’s-Hertogenbosch, the Netherlands) and STK-PamChips^®^ (PamGene International). Each array consists of 140 individual peptide phospho-sites that are analogues of substrates for the corresponding serine-/threonine kinases.

First, whole cell lysates were prepared using M-PER Mammalian Extraction Buffer (Pierce, Waltham, MA, USA) comprising Halt Phosphatase Inhibitor (Pierce) and EDTA-free Halt Protease Inhibitor Cocktail (Pierce). Next, a mixture of 1 µg of total extracted protein and 400 µM ATP was prepared and applied per each array. During the reaction anti-phospho-Ser/Thr antibodies bind sequence-specific peptide phosphorylation followed by detection via a secondary polyclonal swine anti-rabbit Immunoglobulin-FITC antibody (PamGene International). A CCD camera and the Evolve software v. 1.0 (PamGene International) were used to record the signal and the quality of signals was controlled. For the data analysis a log2-transformation of final signal intensities was performed and the BioNavigator software v. 6.0 (BN6, PamGene International) was used for data analysis und upstream kinase analysis.

### 3.7. Western Blotting

The Western blot assay was executed as previously described [49]: 1 × 10^6^ cells were seeded in Petri dishes (ø 6 cm, 5 mL/dish) and incubated overnight. On the next day, the cells were treated with the compounds in fresh culture media (5 mL/dish) for the indicated time. Next, cells were harvested with a cell scraper and lysed in lysis buffer including protease and phosphatase inhibitors cocktail (Roche, Mannheim, Germany). Cell debris were removed from the protein lysates via centrifugation. Afterwards, the cell-free protein mixtures were separated in gradient ready-made Mini-PROTEAN^®^ TGX Stain-Free^TM^ gels (Bio-Rad, Hercules, CA, USA) using SDS-PAGE (sodium dodecylsulfate polyacrylamide gel electrophoresis). Then, the proteins were blotted onto ø 0.2 µm pore PVDF membrane. The membrane was blocked and consequently treated with primary and secondary antibodies for protein detection. The signals were detected using the ECL chemiluminescence system (Thermo Scientific, Rockford, IL, USA). The antibodies used are listed in Table 2.

### 3.8. Drug Combination Studies

The experiments were carried out as previously reported [46]. In brief, the Zero interaction potency (ZIP) reference model [39] and the online-based SynergyFinder 2.0 tool (https://synergyfinder.fimm.fi, accessed on 20 July 2022 [50]) were used to examine the synergistic or antagonistic cytotoxic effects of NB combined with clinically used cytotoxic drug docetaxel, or MAPK inhibitors PD98059, SB203580, and SP600125. 22Rv1 cells (6 × 10^3^ cells/well in 96-well plates) were simultaneously treated with NB and docetaxel; or pretreated with MAPK inhibitors for 1 h following treatment with NB in the total media volume of 100 µL/well. Following 24 h of incubation, the viability of the cells treated with individual drugs as well as their combinations was determined using MTT assay. The generated data was analyzed and the deviations between observed and expected responses of the different drug combinations are visualized using the SynergyFinder 2.0 software. Red areas (positive δ-values) and green areas (negative δ-values) indicate synergism and antagonism, respectively.

### 3.9. Cell Cycle Progression and DNA Fragmentation Analysis

DNA fragmentation and cell cycle progression of treated and untreated cells (negative control) were analyzed by flow cytometry as previously described [49]. Cells were seeded in 12-well plates (10^5^ cells/well) and incubated overnight. Then, the cells were treated with the indicated concentrations of the tested drugs in 1 mL/well for 48 h. Cells were harvested using trypsin, fixed in 70% ethanol, and stained with propidium iodide. Data on cell cycle phase distributions and DNA fragmentation were collected using FACS Calibur (BD Bioscience, San Jose, CA, USA). Cells appearing as sub-G1 population were considered to contain fragmented DNA.

### 3.10. Annexin-V-FITC/PI Double Staining

Externalization of phosphatidylserine is a distinct marker of apoptotic cell death that can be detected by flow cytometry using annexin-V-FITC and propidium iodide (PI) double staining. The experiment was performed as previously described [49]. In brief, 12-well plates were used to seed 2 × 10^5^ cells/well. After incubation overnight, the cells were pretreated with 100 µM of z-VAD(OMe)-fmk (pan-caspase inhibitor in 1 mL/well), or with the vehicle for 1 h. Next, the tested drugs were added, and the plates were incubated for 48 h. Cells were harvested utilizing trypsin, stained with annexin-V-FITC and propidium iodide, and analyzed using FACS Calibur (BD Bioscience, San Jose, CA, USA).

### 3.11. P-Glycoprotein Activity Analysis

The experiment was performed as previously described [42]. In brief, 6 × 10^3^ cells/well of the p-gp overexpressing PC3-DR cells were seeded in black clear bottomed 96-well plates in docetaxel-free culture medium (100 µL/well). The plates were incubated overnight and the media was replaced with the DPBS (50 µL/well) containing investigated drugs at various concentrations. The plates were then incubated for 30 min. Afterwards, 50 µL of 1 µM of calcein-AM solution in DPBS were added into each well and the plates were incubated for another 15 min. The green fluorescence was measured with Infinite F200PRO reader (TECAN, Männedorf, Switzerland). The values were normalized to the possible background autofluorescence of the drugs’ solutions.

### 3.12. Data and Statistical Analysis

All experiments were performed in triplicates (*n* = 3, biological replicates) unless otherwise stated. Cells treated with vehicle were used as a control. Calculations of IC_50_s as well as statistical analysis were performed using GraphPad Prism v.9.1.1 software (GraphPad Software, San Diego, CA, USA). Data are represented as mean ± standard deviation (SD). The Student’s *t*-test was used for comparison of two groups; the one-way ANOVA followed by Dunnett’s post-hoc tests were used for comparison of multiple groups. Statistically significant difference is indicated with asterisk (*) if *p* < 0.05 in both the ANOVA or Student’s *t*-test.

## 4. Conclusions

In conclusion, NB revealed promising anticancer activity in various prostate cancer cell lines harboring different levels of drug resistance. The compound was active in docetaxel-resistant PC3-DR cells and in hormone-independent 22Rv1 cells. Anticancer activity was, at least in part, executed by the induction of caspase-independent cell death. Despite having a moderate p-gp substrate-like affinity, NB did not exhibit cross-resistance to docetaxel in p-gp-overexpressing PC3-DR cells. In contrast, the compound was found to be a potent p-gp inhibitor and could resensitize the cells to docetaxel. An analysis of its effects on the cancer cell kinome revealed NB to induce mitogen-activated protein kinases JNK1/2 and p38. Further functional analyses confirmed the activation of both kinases and indicated a prosurvival role of these biological events in the cellular response to NB treatment. Overall, NB holds a promising anticancer potential, although further studies and structural optimization would be needed in order to improve its anticancer properties. In addition, an inhibition of the above-reported resistance mechanisms should be considered to further increase the effect of NB.

## Figures and Tables

**Table 1:** Selectivity and cytotoxicity of the NB in prostate cancer cells. IC_50_s were determined after 48 h of treatment using MTT assay. The values are represented as mean ± SD. Selectivity index (SI) was calculated as follows: [mean IC_50_ in noncancer cells]/[mean IC_50_ in cancer cells]. Docetaxel was used as a reference drug.

Compound	IC_50_ [µM]										Mean IC_50_, Cancer Cells	Mean IC_50_, Noncancer Cells	Selectivity Index (SI)
	Prostate Cancer Cells						Noncancer Cells						
	PC3	PC3-DR	DU145	22Rv1	VCaP	LNCaP	PNT2	RWPE-1	HEK293	MRC-9			
**NB [µM]**	6.03 ± 1.18	3.63 ± 1.03	12.97 ± 2.69	3.02 ± 2.5	0.143 ± 0.145	12.15 ± 2.97	25.13 ± 5.92	16.4 ± 1.87	9.3 ± 1.57	11.12 ± 4.3	6.46	15.49	2.4
**Doce-taxel [nM]**	7.49 ± 7.09	324.9 ± 21.7	2.2 ± 0.6	0.6 ± 0.12	0.23 ± 0.22	5.06 ± 0.58	>500	0.42 ± 0.23	11.49 ± 3.22	>500	56.75	252.98	4.5

**Table 2:** List of antibodies used.

Antibodies	Clonality	Source	Cat.-No.	Dilution	Manufacturer
anti-ERK1/2	mAb	mouse	#9107	1:2000	Cell Signaling
anti-JNK1/2	mAb	rabbit	#9258	1:1000	Cell Signaling
anti-p38	mAb	rabbit	#9212	1:1000	Cell Signaling
anti-phospho-ERK1/2	mAb	rabbit	#4377	1:1000	Cell Signaling
anti-phospho-JNK1/2	mAb	rabbit	#4668	1:1000	Cell Signaling
anti-phospho-p38	mAb	rabbit	#4511	1:1000	Cell Signaling
anti-β-Actin-HRP	pAb	goat	sc-1616	1:10,000	Santa Cruz
anti-MDR1 (p-gp)	mAb	rabbit	#13342	1:1000	Cell Signaling
anti-α-Tubulin	mAb	mouse	T5168	1:5000	Sigma-Aldrich
anti-LC3B-I/II	pAb	rabbit	#2775	1:1000	Cell Signaling
anti-cleaved Caspase-3	mAb	rabbit	#9664	1:1000	Cell Signaling
anti-PARP	pAb	rabbit	#9542	1:1000	Cell Signaling
anti-Survivin	pAb	rabbit	NB500-201	1:1000	Novus
anti-mouse IgG-HRP		sheep	NXA931	1:10,000	GE Healthcare
anti-rabbit IgG-HRP		goat	#7074	1:5000	Cell Signaling

## Data Availability

The original data are available from the correspondent authors on request.
